# Eulogy of Rudolf Nieuwenhuys

**DOI:** 10.1007/s00429-025-02929-1

**Published:** 2025-05-21

**Authors:** Katrin Amunts

**Affiliations:** 1 Institute of Neuroscience and Medicine, Structural and Functional Organization of the Brain (INM-1), Research Centre Jülich GmbH, Jülich, Germany; 2 C. and O. Vogt Institute for Brain Research, University Hospital Düsseldorf, Heinrich Heine University, Düsseldorf, Bundesrepublik Deutschland

Eulogy of Rudolf Nieuwenhuys.

My first contact with the neuroscientist Rudolf Nieuwenhuys goes back to 2000, when I was working on Broca’s region. I came across the concept of the frontal operculum – a region that is often mentioned in the Language literature, but with different anatomical meanings this needs to be Pluralis. His definition was well aligned with our Understanding of the anatomy of this region. Our paths have crossed many times over the years. Rudolf had an incredibly deep knowledge and, at the same time, oversaw a very broad field of research – for example his Immunohistological work, comparative analyses, work on the brain stem and hypothalamus. He provided most detailed reviews for the cerebellum, the insular cortex, and the spine, to name of few of many. This knowledge was reflected in his excellent textbook *The Human Central Nervous System*, which he co-edited with Jan Voogd and Christiaan Van Huijzen (Nieuwenhuys et al., 2008). This book has become a classic and a important part of my teaching at Düsseldorf university, where I am meanwhile leading the Cécile and Oskar Vogt Institute. the most beautiful illustrations make it easy for students to imagine how the human brain is structured and to approach the anatomical details that are described in the text at a high academic level.

Karl Zilles and I invited Rudolf to Jülich in 2013. He gave a talk on the architectonics of the human neocortex and I was deeply impressed by this famous Dutch neuroscientist, who was so familiar with the German school of architectonic brain mapping, in particular with Oskar and Cécile Vogt and Korbinian Brodmann. While Brodmann focused on cytoarchitecture, i.e., the distribution and arrangement of cell bodies, the Vogts studied the distribution and arrangement of axons (Zilles and Amunts 2010). The Vogts pioneered the classical myeloarchitecture as a method of parcellating the cerebral cortex using light microscopy of histological sections stained for myelin (Vogt 1919).

The Vogts’ papers, written in German, are not easy to read, even for German speakers, because of the highly academic style of writing, enriched with a large amount of technical terminology that the Vogts introduced to describe the different architectural types of the cerebral cortex. Rudolf’s German language skills were simply excellent, and he was able to study the original publications.

Based on microscopy and detailed myeloarchitectonic maps, he emphasised the importance of the detailed cortical mapping of Brodmann and the Vogts. As a renowned neuroanatomist and brain researcher, Nieuwenhuys recognised the importance of the Vogts’ myeloarchitectonic studies for understanding the functional organisation of the brain (Nieuwenhuys 2013). He also understood the relevance of myeloarchitecture for modern neuroimaging. However, in order to directly compare Vogts’ maps with results from neuroimaging, it was necessary to find a way to project the original two-dimensional schematic drawings into a three-dimensional space (Nieuwenhuys and Broere 2023). This work was all the more challenging because the Vogts published their myeloarchitectonic maps region-wise in 20 papers.

Nieuwenhuys and Broere (Nieuwenhuys and Broere 2023) have seen the potential of comparing such maps with the multimodal parcellation of the Human Connectome Project (HCP) (Glasser et al., 2016) and the quantitative cyto- and receptorarchitectonic studies integrated into the Julich Brain Atlas (Amunts et al., 2020) developed at our lab. Their paper concluded that “Hopefully, the results of these comparisons will ultimately contribute to the construction of the unified canonical map, which the Vogts had originally in mind”. While the comparison with the parcellation of the HCP resulted in a publication last year (Nieuwenhuys and Glasser, 2024), the comparison with Julich-Brain remains a project of future research.

In several publications, Nieuwenhuys explicitly paid tribute to the work of the Vogts and their co-workers and successors and made their contributions to brain research accessible to a wider audience. By meticulously analysing and integrating the historical data and integrating them into modern neuroanatomical concepts, he helped to keep the Vogts’ legacy alive in the scientific community. Without Rudolf Nieuwenhuys, the knowledge of the myeloarchitecture of the brain and the pioneering work of the Vogts would be less present in modern neuroscience. He made a decisive contribution not only in preserving their findings, but also in developing them further and placing them in the current context of brain research.

## Data Availability

No datasets were generated or analysed during the current study.
