# Correction: Eulogy of Rudolf Nieuwenhuys

**DOI:** 10.1007/s00429-025-02969-7

**Published:** 2025-06-23

**Authors:** Katrin Amunts

**Affiliations:** 1 Institute of Neuroscience and Medicine, Structural and Functional Organization of the Brain (INM-1), Research Centre Jülich GmbH, Jülich, Germany; 2 C. and O. Vogt Institute for Brain Research, University Hospital Düsseldorf, Heinrich Heine University, Düsseldorf, Bundesrepublik Deutschland

**Correction: Brain Structure and Function (2025) 230:70**

10.1007/s00429-025-02929-1

In this article the title was incorrectly given as ‘Katrin Amunts’ but should have been Eulogy of Rudolf Nieuwenhuys’.

The original article has been corrected.

